# A Low-Cost Wireless Bite Force Measurement Device

**DOI:** 10.3390/ma15114000

**Published:** 2022-06-04

**Authors:** Paolo De Pasquale, Erasmo Rubino, Daniele Borzelli, Matteo Peditto, Enrico Nastro Siniscalchi, Francesco Saverio De Ponte, Giacomo Oteri, Andrea d’Avella

**Affiliations:** 1 Department of Biomedical and Dental Sciences and Morphofunctional Imaging, University of Messina, 98100 Messina, Italy; erasmo94@hotmail.it (E.R.); daniele.borzelli@unime.it (D.B.); matteo.peditto@unime.it (M.P.); enrico.nastrosiniscalchi@unime.it (E.N.S.); francesco.deponte@unime.it (F.S.D.P.); giacomo.oteri@unime.it (G.O.); andrea.davella@unime.it (A.d.); 2 Laboratory of Neuromotor Physiology, IRCCS Fondazione Santa Lucia, 00179 Rome, Italy

**Keywords:** 3D printing, biomechanics, bite force, low-cost, masticatory muscles, medical devices, open source, telemedicine, temporomandibular joint, wireless

## Abstract

Assessing maximum voluntary bite force is important to characterize the functional state of the masticatory system. Due to several factors affecting the estimation of the maximum bite force, a unique solution combining desirable features such as reliability, accuracy, precision, usability, and comfort is not available. The aim of the present study was to develop a low-cost bite force measurement device allowing for subject-specific customization, comfortable bite force expression, and reliable force estimation over time. The device was realized using an inexpensive load cell, two 3D printed ergonomic forks hosting reusable subject-specific silicone molds, a read-out system based on a low-cost microcontroller, and a wireless link to a personal computer. A simple model was used to estimate bite force taking into account individual morphology and device placement in the mouth. Measurement reliability, accuracy, and precision were assessed on a calibration dataset. A validation procedure on healthy participants was performed to assess the repeatability of the measurements over multiple repetitions and sessions. A 2% precision and 2% accuracy were achieved on measurements of forces in the physiological range of adult bite forces. Multiple recordings on healthy participants demonstrated good repeatability (coefficient of variation 11%) with no significant effect of repetition and session. The novel device provides an affordable and reliable solution for assessing maximum bite force that can be easily used to perform clinical evaluations in single sessions or in longitudinal studies.

## 1. Introduction

Maximum bite force (MBF) is an indicator of the functional state of the masticatory system [1]. Individual MBF has been used to evaluate jaw muscle functionality and activity and the therapeutic effect of prosthetic devices [2]; it is considered important in the diagnosis of the disturbances of the stomatognathic system. For example, MBF in patients with symptoms of temporomandibular disorders (TMD) is lower than in healthy subjects [3]. However, MBF may vary substantially across healthy subjects because it depends on several anatomical and physiological factors: gender, age, general physical structure (height and weight), cranio-facial morphology, pain, and occlusal factors [4]. MBF estimation is also affected by the mechanical characteristics and measurement technique of the recording device [5]. Indeed, a large variability has been found when recording MBF with different devices [6] and in the sensitivity, i.e., the slope of the load-response curve, of different sensors [7].

Moreover, due to the oral cavity morphology, the biomechanical characteristics of the mouth are not homogeneous, and the position of the force transducer relative to the dental arch also affects MBF, as the more anteriorly MBF is recorded, the smaller is the maximum force achieved. Indeed, from the literature, it is well known that MBF varies in different regions of the oral cavity [8], with different anterior vertical jaw openings [9], and that bilateral clenching is larger than unilateral [10]. As a result of the effects of all these parameters, MBF values reported for the molar region of healthy young adults may vary in a wide range across studies: 113-1692 N [11], 446-1221 N [5], 216-740 N [12], with lower values reported for the incisal region: 108-293 N [11,12,13,14]. A previous study stated that when masseter muscle activity levels were kept constant, MBF varied with bite opening, and the maximum MBF was recorded with an anterior vertical jaw opening between 15 and 20 mm [9]. There has also been disagreement about MBF differences between men and women. In some studies, no difference between genders was detected, whereas, in other studies, men produced greater bite forces than women [11,12,15,16,17].

Several technologies and techniques have been used to record MBF. At present, most of the devices use strain-gauge, piezoresistive, piezoelectric, and pressure-sensitive force transducers. Strain-gauge transducer devices, consisting of a metal plate or fork whose deformation leads to resistance changes, have been proven to be highly sensitive, accurate, and capable of operating with a large measuring range. However, it is still difficult to record a true MBF due to discomfort and the fear of breaking the edges of the teeth when biting the hard surface of the transducer [18,19,20]. Moreover, for the maximal incisal region force recording, “pain in teeth” might be the major limiting factor for expressing MBF [11], and since the surface of the recording devices is usually made with hard materials, an uncomfortable and hazardous feeling may be perceived by many subjects [4]. Placement of the sensor relative to the force application point may also affect measured MBF due to the mechanical leverage caused by the metal plate of the bite fork used in the strain gauge transducer [21,22]. Piezoresistive transducer devices, consisting of a crystal silicone material that changes resistivity with the applied force, have been proven to be highly sensitive, thin, lightweight, and cheap but less accurate than strain gauge devices [21]. Piezoelectric transducers use the piezoelectric effect of a material to convert the pressure into an electric signal. These devices are generally very thin (0.1 mm) and can be used to record MBF in subjects with minimal jaw opening, but they are limited by a narrow range and low sensitivity and flexibility of the sensor [21]. Pressure-sensitive devices consist of a chamber filled with fluid or air and a pressure gauge, which measures chamber pressure. Since the bite element is soft, the advantage of using these devices is that MBF can be recorded safely and comfortably; however, they are less reliable with respect to the other device types [21]. Pressure-sensitive film devices consist of a pressure-sensitive sheet, which changes color according to the applied pressure. Due to their thinness, these devices do not interfere with the occlusion; however, they cannot perform continuous measurements, and they need analytical equipment to analyze the data [21].

One critical open issue concerning MBF recording is that despite several devices and techniques that have been developed, a standardized measurement method that is also easy to use and reliable is still lacking [23]. Most of the developed devices can record force levels in the range of 50–800 N with an accuracy level of 10 N [4,9,18]. Moreover, because of the inherent variability in placing the sensor in the patient’s mouth, highly repeatable measurements of individual bite force are challenging [24], especially in the premolar or molar region [7]. Another important aspect of MBF measurement devices is their cost and complexity. For example, servo-controlled motors or load cells mounted on a customized dental device have been used to investigate motor function and evaluate bite force. However, due to the complexity and costly technical procedure, these approaches are more suited for research purposes than for routine clinical examination [7].

Three-dimensional printing or additive manufacturing is a process for making 3D objects from a 3D model. The technology consists of an additive process in which successive layers of material are laid down under computer control. Nowadays, thanks to the development of affordable 3D printing technologies, rapid prototyping using 3D printers has become widespread and has a wide range of applications in several fields, such as research engineering and the medical industry. An important advantage of 3D printing is the ease of manufacturing any object of any shape with the same characteristic in any part of the world from a 3D model as the stereolithographic STL file format, which is the standard format currently used for 3D printing [25].

The aim of the present study was to develop a novel device for the estimation of the maximum bite force that could be built in-house at a low cost and would allow recording multiple standardized incisal maximum bite forces with adequate reliability and repeatability. A comparative analysis of several force sensors, estimation techniques, and maximum expression influential factors led to the selection of the most suitable force transducer for high range reliable measurement, the design of a sensor interface allowing to express MBF in the most physiological jaw separation, and the development of methods allowing the standardization and repeatability of the measurements across multiple experimental sessions and subjects, taking into account subject’s morphology. Safe operation characteristics, cost effectiveness, ease of use, and the possibility to build it in-house make the device a useful tool for investigating the masticatory system functionality of adults in a clinical and non-clinical setting for diagnosis and/or monitoring of the therapy of patients with muscular and/or orthopedic TMD and also in affordable telemedicine scenarios. The open-source software and CAD designs are made freely available to enable easy replication of the device.

## 2. Materials and Methods

**Figure 1:**
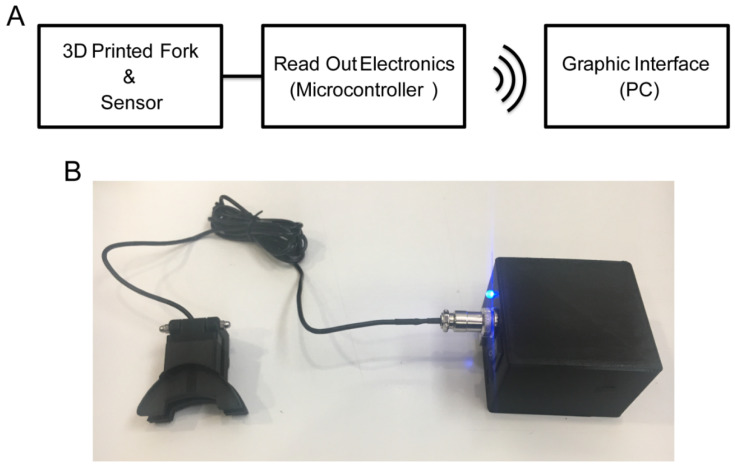
Bite force measurement device. (**A**) Schematic representation of the system: the force sensor placed in a 3D printed forks interface is wired connected to the electronic instrumentation, which is wirelessly connected to the PC. (**B**) Developed device: the force sensor with the 3D printed interface connected to the electronic instrumentation (box).

A bite force measurement device consisting of a force sensor, placed in between two 3D printed ergonomic forks and connected to a read-out system interfaced through a wireless link to a personal computer was developed (PC) (Figure 1). A reusable silicone mold, customized for each participant, provides the interface between the forks and the subject’s teeth. Thanks to the mold interface and to an offline procedure, it is possible to estimate the device placement within the oral cavity and the distance of the force transducer from force application points. A model was developed to describe the physical interactions between the masticatory system and the device. A calibration procedure was implemented, and three sets of data were collected with both the device and an accurate industrial force transducer as reference. A linear regression analysis was performed on the first dataset to calibrate the device. The reliability of calibration parameters was then assessed using the other two datasets. A validation procedure with multiple recordings was performed on 16 healthy participants. Precision over multiple sessions and repetitions across experimental conditions was analyzed using linear mixed models (LMM). For each participant, the coefficient of variation of the recorded MBF values recorded during all the repetitions within the three sessions was used as an indicator of individual variability.

### 2.1. Force Sensor

A small (20 × 11 mm) and inexpensive alloy steel load cell (TAS606, Ht Sensor Technology Co., Ltd., Xi’an, China) capable of measuring up to 2000 N was used to measure bite force. The load cell transducer has four strain gauges connected in a Wheatstone bridge formation, allowing to measure changes in resistance with an accuracy of 0.3% of the full scale.

### 2.2. Custom Made Ergonomic Design and In-House 3D Printing

**Figure 2:**
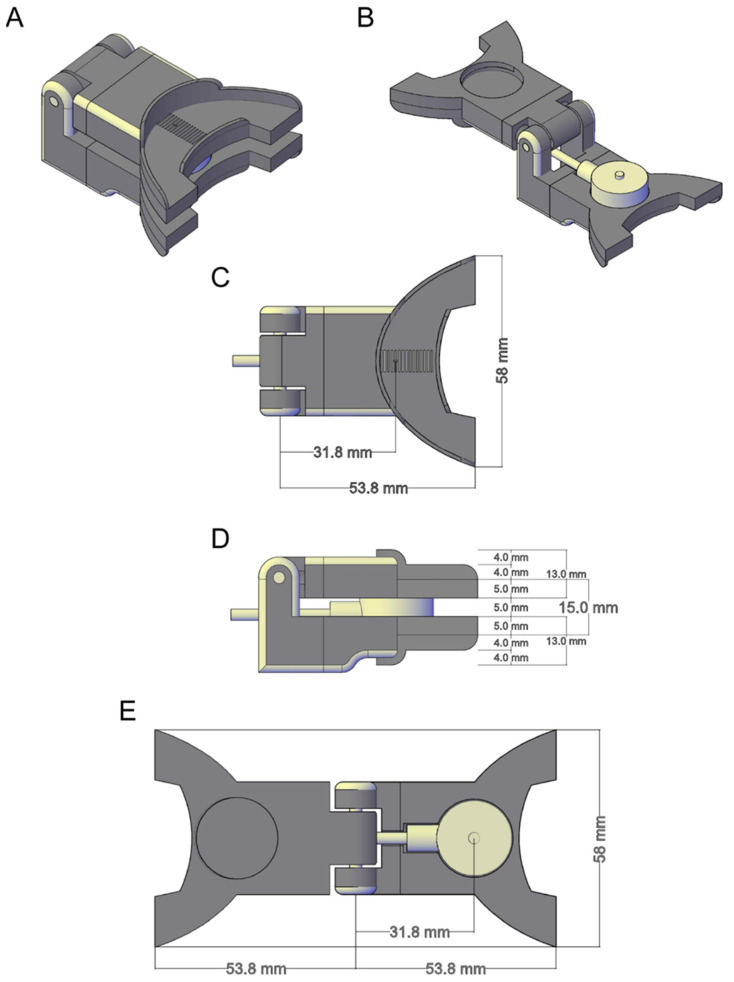
Interface of 3D CAD model. Representation of the 3D printed interface and the force measurement sensor CAD models. (**A**,**B**) Isometric view of the 3D sensor placed in the interface CAD while the upper fork plate is closed (**A**) and open (**B**). (**C**,**D**) The top-bottom and medio-lateral views of the sensor-interface CAD with the relative sizes, respectively. (**E**) Top-bottom view of the sensor while the upper fork plate is open.

A custom-made ergonomic interface between the sensor and the participant’s teeth was designed with commercial CAD software (AutoCAD, Autodesk Inc., San Rafael, CA, USA) and printed with a 3D printer (Supplementary Materials CAD drawing and stereolithography meshes S1). The interface consists of two hinged elements with a slot for inserting the load cell (Figure 2). Each element (maximum length 53.8 mm) had the shape and size of a medium bite fork for dental records. At a distance of 31.8 mm from the hinge axis (fulcrum) is the center of a circular slot for the insertion of the force sensor. At the edges of each fork plate, a series of vertical flanges have been 3D printed to contain the silicone mold and to guide its repositioning. A series of notches placed at 1 mm steps, starting from the center of the force sensor, indicated with a cross notch, were printed on each fork plate. When the mold is placed on the fork plate and pressure is exerted, these notches leave an indentation on the mold. An offline procedure then allows measuring the distance between the center of the sensor (cross notch) and the incisal teeth by comparing the position impressed on the mold of the incisal teeth with respect to the indentations generated by the notches. The total vertical distance between the force application points (on the upper surface of the top element and on the lower surface of the bottom element) was 15.0 mm with the force sensor inserted between the two elements. A commercial 3D printing machine (Ultimaker 2 Extended+, Ultimaker B.V., Geldermalsen, The Netherlands) was used for prototyping the device. Polylactic Acid (PLA) was chosen as the printing material because it is biodegradable and non-toxic when used in solid form. A nozzle of 0.4 mm was used to lay down, at a speed of 50 mm/s, a layer of material with a grid infill pattern and a density of 20%. Stereolithography meshes used for 3D printing can be found in the Supplementary Materials CAD drawing and stereolithography meshes S1.

### 2.3. Read-Out System

The data read-out system consisted of a microcontroller (Arduino UNO, Arduino S.r.l.) connected to the load cell through an amplifier (HX711, AVIA Semiconductor), powered by a 3.7 V lithium-ion battery with 2000 mAh capacity. A Bluetooth module (HC-05, iTeadStudio) is used to establish a wireless link to transmit data from the microcontroller to a PC and to control the device from the PC. Read-out components are mounted in a custom-made 3D printed housing. The read-out components and circuitry scheme can be found in Supplementary Figure S1, and the 3D CAD model and the stereolithography meshes used for 3D printing the housing can be found in Supplementary Materials CAD drawing and stereolithography meshes S1.

### 2.4. Software

**Figure 3:**
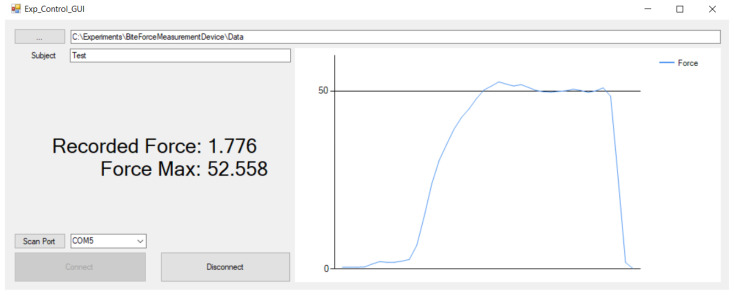
Data visualization software interface. C# based interface running on a PC allows visualizing and saving data sent wirelessly from the Arduino board. The software interface displays the instantaneous expressed force (Kg) and MBF achieved during the whole session.

Software modules for data collection, device control, and data display were developed using two different open-source solutions. The software running on the Arduino microcontroller collects data from the force measurement sensor and transmits it via Bluetooth to the PC, which is implemented using Arduino scripting language. The graphical user interface controlling data collection and display on the PC (Figure 3) was developed in C#. Arduino sketches and the PC GUI C# Visual Studio project can be found in Supplementary Materials Software S1.

### 2.5. Physical Model

**Figure 4:**
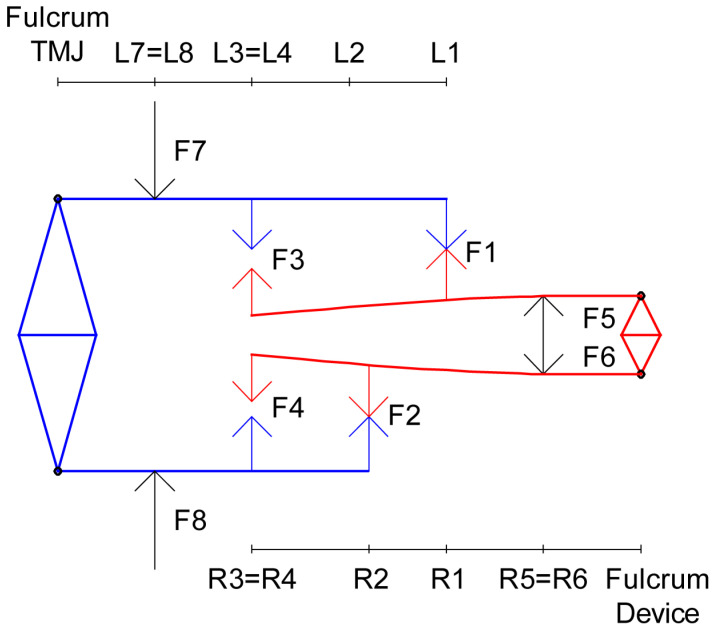
Schematic representation of masticatory system (blue) and device (red) model system. *F*7 and *F*8 are equal and opposite forces exerted by the masticatory muscles; *F*5 and *F*6 are forces recorded by the sensor. For simplicity, only two contact points between the mouth and the device are considered for each arc: *F*1 and *F*3 for the upper part, *F*2 and *F*4 for the lower one.

Since the position of the measuring sensor with respect to the dental arch is necessary to measure MBF, the point of force application by the dental arches on the fork plates must be estimated. A model (Figure 4), consisting of a simplified representation of both the masticatory system (blue) and the device (red), allowed us to characterize the mechanical interaction between the fork plates and the dental arches and to determine that the force application point occurs at the level of the incisors. In particular, the force recorded by the force sensor on the device is proportional to the force exerted by the incisors furthest away from the force sensor.

The device is modeled as a second-order lever with the load (force transducer) between the fulcrum and the effort (bite force). The masticatory system can be described as a third-class lever with the effort (masticatory muscles force) between the fulcrum (condyle) and the load (teeth-device application points). The force recorded on the transducer is proportional to the force applied by the masticatory muscles. In particular, since the device acts as a lever, the applied force can be estimated from torque balance and depends on the difference between the distance from the sensor to the fulcrum and the distance from the force application point to the fulcrum, which are the lever arms. Moreover, since the device is a second-order lever, the difference between lever arms will provide a mechanical advantage (gain) in the recorded force with respect to the real one. When the mouth is open, the forces applied by the masticatory muscles (Figure 4, *F*7, and *F*8, equal and opposite forces) rotate the jaws around the fulcrum. For simplicity, only two contact points between each dental arch and each element of the device are considered: *F*1 and *F*3 for the top element, *F*2 and *F*4 for the bottom element. However, the method can be generalized to an arbitrary number of contact points without affecting the result. The problem can be considered as a statically indeterminate problem in which the laws of static are not sufficient to determinate all the unknown forces or moments [26]. This problem can be solved by writing the appropriate equations of static equilibrium and additional equations pertaining to the deformation and constraints known as compatibility conditions (see Appendix A). From the characterization of the device, if the distance *R*1 between the application point of *F*1 and the fulcrum of the device is greater than the distance *R*2 for *F*2, then the sensor momentum (*Ms* = *Fs*·*Rs*) is equal to the momentum at the upper incisal teeth (*M*1 = *F*1·*R*1) because the top element of the device works as a hyperstatic beam and the force *F*3 is equal to zero. Conversely, if *R*1 is smaller than *R*2, then *Ms* is proportional to the momentum at the upper lower incisal teeth (*M*2 = *F*2·*R*2), and *F*4 is equal to zero, see Equation (1). Therefore, the force recorded by the sensor depends on the distance between the sensor and the most distant incisal application point.

(1) $$\{\begin{array}{c} F1=\frac{Fs\cdot Rs}{R1}\;,\;\;if\;R1>R2\\ F2=\frac{Fs\cdot Rs}{R2}\;,\;\;if\;R1<R2 \end{array}$$

### 2.6. Calibration Procedure Using a Second Force Transducer

**Figure 5:**
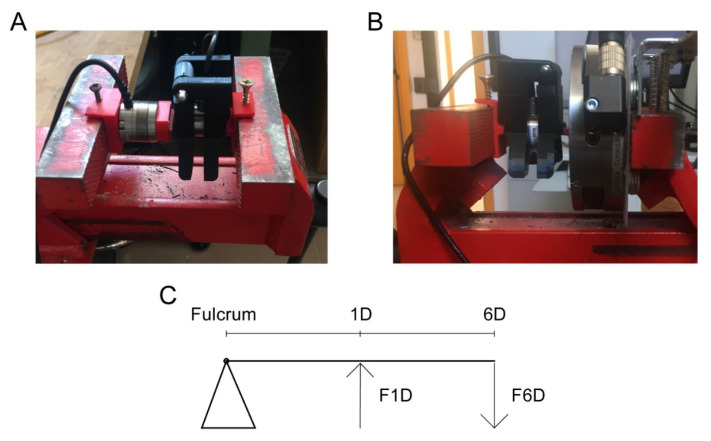
Calibration procedure. A manual press machine applies several loads on the device through a second calibrated load cell. (**A**) The calibration procedure used a small 6D force transducer (max force 500 N) as the second device; (**B**) the setup with a large 6D force transducer (max force 495 N). (**C**) Schematic representation of the second-order lever, which characterizes the calibration process. In particular, the press machine exerts a force on the 6 DOF (*F*6*D*) sensor, which can be placed at a variable distance (∆*d* = *d*_6*D*_ − *d*_1*D*_) from the main axes of the 1 DOF. *d*_6*D*_ and *d*_1*D*_ are, respectively, the distances from the fulcrum of the lever of the 6 DOF and 1 DOF sensors. *F*1*D* is the resulting force recorded on the 1 DOF sensor.

To calibrate the device, it was loaded with known forces through a manual press. To measure the forces applied by the press, two accurate 6-axis force transducers were used: a small 6-axis transducer (Nano 25 F/T Sensor, ATI Industrial Automation, Apex, NC, USA, Figure 5A) calibrated by the manufacturer, with a resolution of 1/16 N and a maximum force of 500 N (single-axis overload: ±7300 N) for the longitudinal axes and a large 6-axis transducer (Delta F/T Sensor, ATI Industrial Automation, Apex, NC, USA, Figure 5B), with a resolution of 1/16 N and a longitudinal maximum force of 495 N (single-axis overload: ±10,000 N) for the longitudinal axis. Forces were applied on the two elements of the device through a 3D-printed PLA interface (base 0.2 × 2 mm) mounted on the 6-axis force transducer so that both devices were firmly coupled with the manual press machine (Figure 5A,B). The system, 1D-6D load cell (Figure 5C), can be characterized by the linear equation derived from the system of torque balance equations [27]:

(2) $$F_{1D}d_{1D}=F_{6D}d_{6D}$$

where *F*_6*D*_ is the force measured by the 6-axis sensor, *F*_1*D*_ is the force measured by the device 1-axis sensor, *d*_1*D*_ = *Lever* is the lever arm, i.e., the distance from the center 1-axis sensor to fulcrum; *d*_6*D*_ = *Lever* + ∆*d* is the distance from the center of 6-axis sensor to the fulcrum:

(3) $$F_{6D}=\frac{F_{1D}Lever}{Lever+\Delta d}=F_{6D}+\frac{F_{6D}\Delta d}{Lever}$$

If *F*_6*D*_ = *x* and *F*_1*D*_ = *y*,

(4) $$y=x+\frac{x\Delta d}{Lever}=x\left(1+\frac{\Delta d}{Lever}\right)$$

(5) $$\hat{y}=Offset+x\;Slope+x\;Slope\frac{\Delta d}{Lever}$$

(6) $$\hat{y}=\beta_{1}+x\;\beta_{2}+x\;\beta_{3}\Delta d$$

where *β*_1_ = *Offset*, *β*_2_ = *Slope*, *β*_3_ = *Slope*/*Lever*. Then:

(7) $$\hat{x}=\frac{y-\beta_{1}}{\beta_{2}+\beta_{3}\Delta d}=\frac{y-Offset}{Slope+Slope\frac{\Delta d}{Lever}}$$

(8) $$\hat{F_{6D}}=\frac{F_{1D}-\beta_{1}}{\beta_{2}+\beta_{3}\Delta d}=\frac{\left(F_{1D}-Offset\right)}{Slope+Slope\frac{\Delta d}{Lever}}$$

The parameters *β*_1_, *β*_2_, and *β*_3_ (Equation (6)) were estimated with a linear regression performed on the dataset. In the first dataset with the small transducer, 21 different force values, ranging from 0 to 490 N (0–50 Kg), were recorded. Each measurement consisted of 50 samples. Then, 2 additional datasets were recorded to test the reliability of the estimated parameters. The first test dataset consisted of 15 force recordings, with a load range from 98 to 490 N with a step size of 100 N and different force application points at 0, 5, and 10 mm from the 1D load cell axis, in order to characterize the lever gain. The second dataset collected with the large transducer consisted of 6 recordings (50 samples each), from 0 to 980 N.

### 2.7. Validation Procedure with Multiple Force Recordings of Healthy Participants

**Figure 6:**
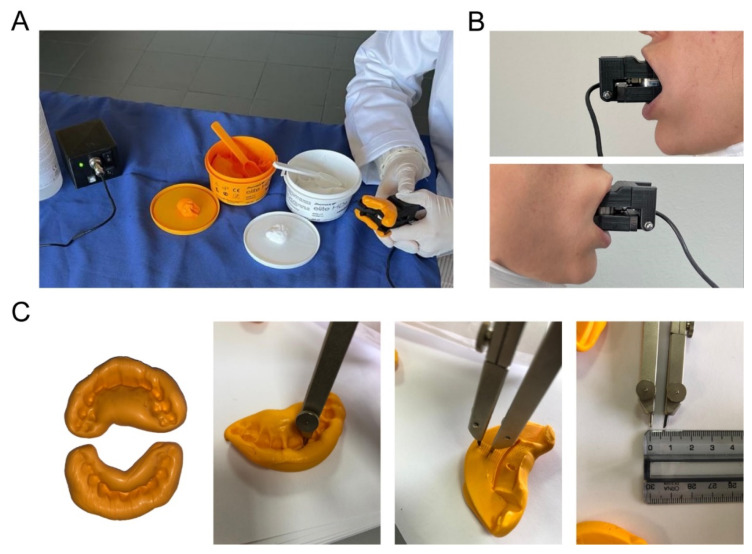
Experimental procedure. (**A**) Device setup steps. During setup phase, therapist places the teeth silicone molds on the device forks. (**B**) Device placed in subject’s mouth. (**C**) Example of the subject’s customized upper and lower silicone molds and the application point distance measurement phase. During this phase, the distances between both incisors, upper and lower, and the center of the sensor are estimated thanks to the notches on the silicone mold.

Sixteen healthy participants (7 females), aged from 24 to 55 years (32 ± 10, mean ± SD), height 155 to 187 cm (170 ± 10 mean ± SD), weight 52 to 87 kg (68 ± 11 mean ± SD), performed 3 consecutive MBF measurement sessions for 3 days. Subjects were tested at the U.O.C. Odontoiatria ed Odontostomatologia of the Azienda Ospedaliera Universitaria (AOU) Gaetano Marino in Messina, were informed of the purposes of the measurement and gave their consent to the measurement and to the collection of personal data. The study was conducted in accordance with the Declaration of Helsinki, and since the measurements did not involve any intervention, they did not require ethical approval according to the standard procedures of the AOU. Subjects were seated on a chair with heads positioned so that the Frankfort horizontal plane would be parallel to the floor while performing the task. When the subject performed the task for the first time, the forks were filled with silicone teeth mold and covered by a plastic thin film that sealed the device from liquids (Figure 6A). The operator placed the device in the mouth of the participant simulating the teeth molding procedure in order to obtain a subject-specific interface (Figure 6B,C). Thanks to the fork supports, the silicone mold can be easily removed from the fork and replaced in the same position for multiday recordings. The silicone mold provides a measure of the incisive teeth application point, which can be used offline in order to estimate the correct MBF value (Figure 6C).

### 2.8. Statistical Analysis

The dependence of MBF on experimental factors was analyzed with a linear mixed model (LMM) that accounts for interindividual variability by including the participant as a random effect. The session (*S*) and the repetition (*R*) within each session were treated as fixed effect factors. Data were fitted with the model described in Equation (9).

(9) $$Y=u_{0}+\alpha_{0}S+\beta_{0}R+\epsilon$$

In Equation (9), *u*_0_ represents the individual intercept and accounts for inter-individual differences. The coefficients *α*_0_ and *β*_0_ represent fixed-effects; thus, the modulation of the response variable by the main factors *S* and *R*. The estimation of model parameters was based on the maximum likelihood approximation. To test the significance of each fixed effect term in the selected model, a hypothesis test on the fixed effect terms applying analysis of variance (ANOVA) on the fitted LMM was performed. The analysis was implemented in Matlab.

## 3. Results

### 3.1. Calibration: Accuracy and Precision

**Figure 7:**
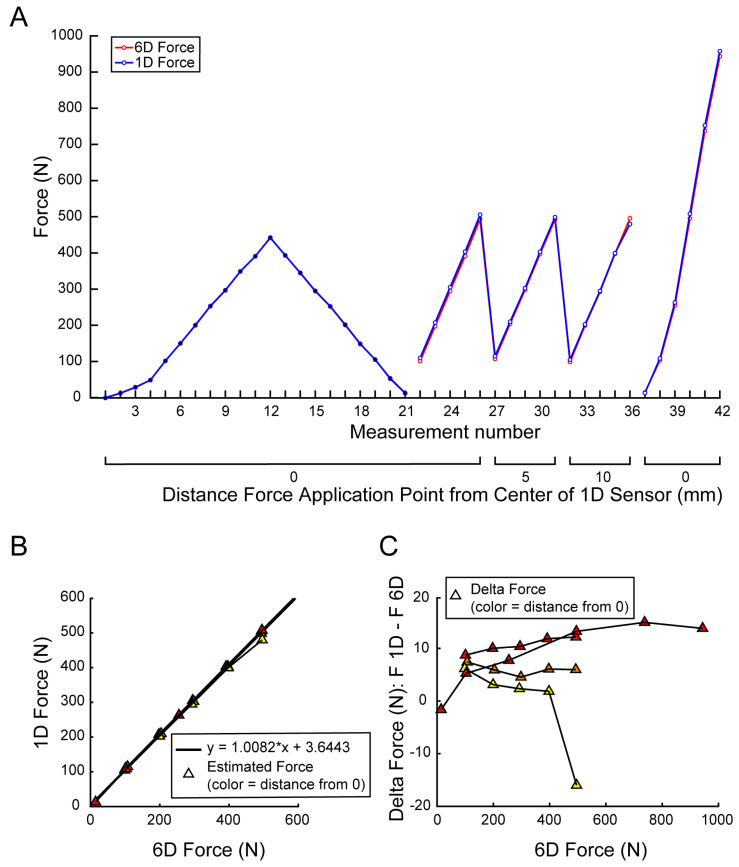
Calibration results. (**A**) Calibration and test session curves. Estimated force recorded with the 6D load cell (red), and the estimated force recorded with the 1D sensor (blue), are reported in the function of the application point distance from the center of the 1D load cell. Black dots are the records used for the calibration; white dots are the data used for the test. (**B**) Compares the 1D load cell estimated force with the 6D load cell one. (**C**) Delta force (1D − 6D estimated force) in function of the 6D one. Triangle color indicates the distance of the force application point from the center of the 1D sensor (yellow: 10 mm; orange: 5 mm; red: 0 mm).

In Figure 7A, the forces recorded during the calibration and testing phases are reported for three datasets. For each estimated point, which represents the mean value over 50 samples, the distance of the force application point from the center of the device (1-axis) force sensor is reported. The forces estimated with the 6-axis sensor (red) and the one recorded with the 1-axis sensor (blue) are reported for the calibration session (filled circular markers) and the test sessions (empty circular markers). The mean force estimation error of the force measured with the device (1-axis sensor) with respect to the 6-axis sensor was 0.00 ± 0.65 N for the calibration dataset and 6.4 ± 6.7 N for the test datasets. Figure 7B shows the force estimated by the device (1-axis sensor) as a function of the force estimated with the accurate 6-axis sensor, and Figure 7C the force residual, for the test sessions. The color of the triangular markers indicates the distance of the force application point from the center of the 1-axis sensor.

### 3.2. Validation: Repeatability and Effect of Session and Participant

**Figure 8:**
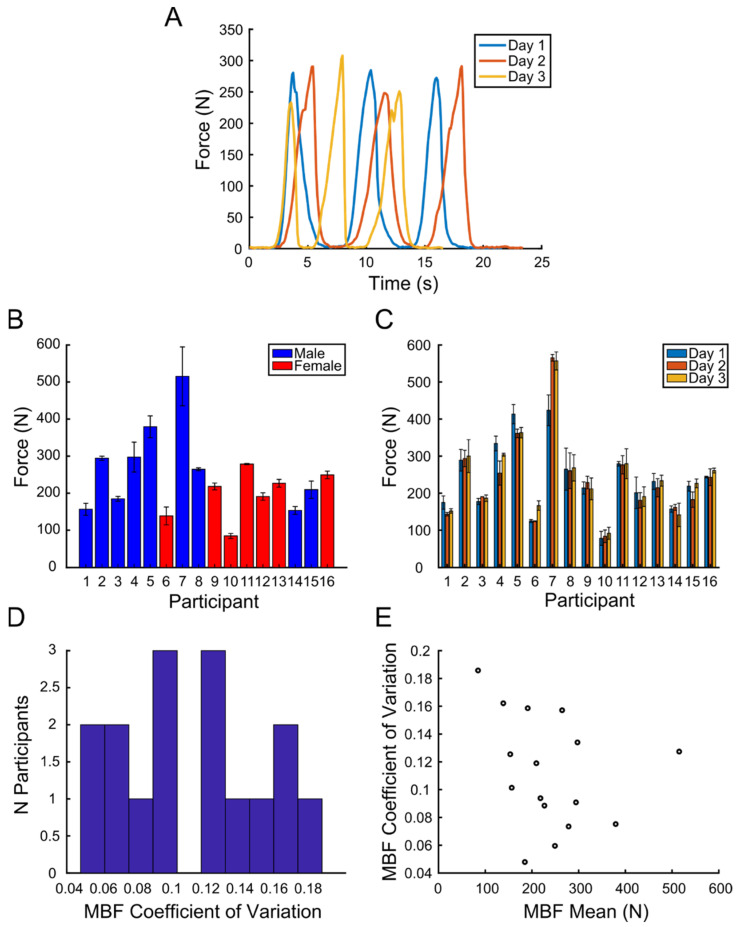
(**A**) Recorded MBF profiles as a function of time for the 3 different sessions (different colors). During each session, subject performed MBF 3 times. (**B**) Mean MBF value over sessions for each subject, (blue) for male and (red) for female. (**C**) Mean MBF value over session repetitions for each subject. (**D**) Histogram of MBF coefficient of variation value, (**E**) MBF coefficient of variation in function of MBF mean.

Figure 8A illustrates an example of the force data recorded in one participant (12). In each recording session (different colors) performed on three different days, the participant generated MBF three times in the course of about 20 s. The average MBF over all sessions for each participant varied from a minimum of 65 N to a maximum of 584 N (see Figure 8B). Gender is indicated with different colors (blue) for male and (red) for female participants. Thus, the data revealed a large inter-individual variability of MBF. The average MBF over participants was 240 ± 105 N (mean ± SD), which is compatible with the mean incisal range found in the literature (108-293 N) [12]. The mean maximum bite force for men was (277 ± 114 N), with the range of 584 to 105 N. The mean maximum bite force for women was (195 ± 64 N), with the range of 308 to 65 N. Figure 8C shows the mean values over repetitions in each session, better highlighting the repeatability over repetitions and sessions. A linear mixed effect (LME) model, with gender, repetition, and session as fixed effects and subject as random effect, did not reveal any statistically significant differences between gender (*p* = 0.25), repetitions (*p* = 0.26), and sessions (*p* = 0.24). Figure 8D shows a broad distribution of the coefficient of variation (CV) of MBF across sessions for each participant. The average CV over participants was 11 ± 4%, indicating a low extent of variation for the recorded forces for each subject, which confirm the precision and repeatability of the measurement within and between sessions. Figure 8E shows the individual CV as a function of MBF. No significant relations between MBF and the CV were found.

## 4. Discussion

Evaluation of MBF is important to assess the functional state of the masticatory system. Despite the various devices using different technologies that have been developed to measure MBF, there is still no standardized measurement method that is also easy to use and reliable. It is well known that the mechanical characteristics and the measurement technique of the recording device can influence the accuracy and precision of MBF estimation [5]. Moreover, as MBF can be affected, in addition to the used technologies, by the presence of TMJ disorders [3], patient-specific anatomical factors [4], and force application point [21,22], device placement within the oral cavity must be standardized and evaluated to reliably estimate MBF. Finally, to obtain adequate reliability and repeatability while performing multiple measurements in the same patient, it is critical to minimize the dependence of the results from the operator and to make an accurate repositioning of the device that is easy to perform.

A novel, low-cost MBF measurement device, based on a commercially available strain gauge sensor, with a nominal working range adequate for use with adult subjects, inserted in a custom housing interface, using a simple microcontroller-based data read-out system connected wirelessly to a data acquisition and display software on a personal computer was introduced. The soft polymeric housing of the sensor can be easily reproduced, without any industrial machinery, with a commercial 3D printer and provides a homogeneous soft surface to bite, which overcomes the fear of breaking edges of the teeth that may occur when biting in the hard surface of the strain-gauge force sensor [18,19,20]. The developed measurement procedure, based on the usage of a reusable subject-specific silicone mold, allows for customizing the device according to individual anatomical factors and easily repositioning the device within the mouth. A series of notches in the 3D printed forks are imprinted by pressure on the silicone mold, providing a graduated indicator that allows to easily estimate the distance between the force sensor and both the upper and lower incisive teeth region. Such estimation is necessary to evaluate the force application point, which may vary across individuals since the distances between both teeth regions and the sensor depend on the anatomical structure. The distance between the sensor and the application point is required for a reliable estimation of MBF since the device acts as a lever due to the developed 3D printed interface design. Neglecting the distance between the device and the force application point when measuring MBF may lead to unreliable force estimation, especially for strain gauge-based devices with a metal fork acting as a lever [21,22]. To reliably estimate MBF according to the participant’s morphology, a model of the interaction between the masticatory system and the device was developed. Then, a test calibration was performed in the laboratory to assess the reliability and repeatability of the device within the physiological range of loads and application points. The device demonstrated satisfactory performance in terms of accuracy and precision in an adequate force range. In particular, the device could record forces in the range of 0-980 N with an accuracy of about 6.4 N and a precision of 6.7 N, corresponding to a mean relative accuracy of 2% over the test dataset and a relative precision of 2% for the mean force value of the test dataset. Previous reviews reported a mean accuracy of 2% in the range 0-350 N [5] or an accuracy of 10 N and 20% relative precision (assuming that one minus relative precision, i.e., 80%, was reported) in the range of 50–800 N [4,9,18]. It is worth noticing that the selected force transducer allows for an even wider force range, up to about 2000 N, but the calibrated load range was adequate for incisive region MBF [11,12,13,14]. The consistency and accuracy of the bite force recorder were further supported by testing 16 adult subjects. As shown in the results section, the reliability and validity of MBF estimations (240 ± 105 N) are in line with published studies using state of the art bite force recording devices for measuring maximum incisal teeth bite force in healthy subjects (108-293 N) [11,12,13,14]. The values recorded were just above those reported in the literature, possibly due to physiological factors specific to the selected participants or to the more comfortable bite afforded by the developed device, which allows for exerting MBF without fear. Differences between gender were also investigated. While in some studies, no differences between gender were detected, in most studies, men produced greater bite forces than women [11,12,15,16,17]. Even if, in our study, men’s mean maximum bite force (277 ± 114 N) was greater than for women (195 ± 64 N), no statistically significant differences in bite force were found [12]. This might be due to the small number of subjects included in the study.

The customization of the device using a subject-specific silicone mold allows for adequate repeatability across multiple sessions. The developed methods also allow performing multiple recordings either within the same day or on different days with high precision (CV of 11 ± 4%) and no statistically significant differences within or across sessions. Because teeth shape may vary and because of the variation in positioning the sensor in the patient’s mouth, which might also be due to operator inaccuracy, force estimation [21,22] may be unreliable [14,24], and highly repeatable measurements of patient MBF may be difficult to achieve [24], especially in the premolar and molar region [7]. However, one study did not find statistically significant differences between repetitions [4], possibly because the authors were very careful in repositioning the sensor within the oral cavity. Since in our device, after the first application, the replacement within the oral cavity is standardized by the silicone mold, the device can be repositioned precisely and also used without the operator’s supervision.

Finally, the design of our device has additional desirable features. To ensure the safety of the device, the electronic read-out component transmits wireless data to a PC for storage, and it is therefore intrinsically safe, as it does not require a power isolation thanks to a low voltage battery supply. Moreover, the device is low-cost, can be easily reproduced, and is able to assess MBF in clinical or domestic settings for characterizing the functionality of the masticatory system for patients with TMJ disorders during a longitudinal study or a rehabilitation program. However, the device also has some limitations. Even if the device is wireless, it requires a PC to visualize and store data. Future developments will address this limitation by integrating a display and a data storage system directly into the small-size, portable read-out system. Another limitation is the requirement of an initial silicone mold customization procedure to standardize device replacement within the mouth, compared to a simple stick fork that can be directly bitten. However, the silicone mold has several benefits, such as the accuracy of the repositioning and the device customization with respect to the patient’s mouth, that justify its use. Moreover, the material required for the mold is easily available in dentistry. Finally, to be reproduced, the device requires a 3D printer. However, nowadays, 3D printing is very affordable.

## 5. Conclusions

A novel computer-assisted design for an MBF measurement device was developed that is portable, cost effective, and open-source. The device is easy to use, reliable and can be employed in both clinical and domestic environments, for accurate functional assessments, for monitoring of the therapy of patients with muscular and/or orthopedic TMD, and in telemedicine scenarios. Further studies will use the novel device to characterize the changes in MBF and evaluate the efficacy of physical therapy in specific pathologies, such as TMD [28] and myotonic dystrophy [29]. Moreover, further development will concern the integration of a low-cost EMG system to study the relation between MBF and myoelectric signals recorded from jaw-closing muscles [9].

## Figures and Tables

**Figure A1:**
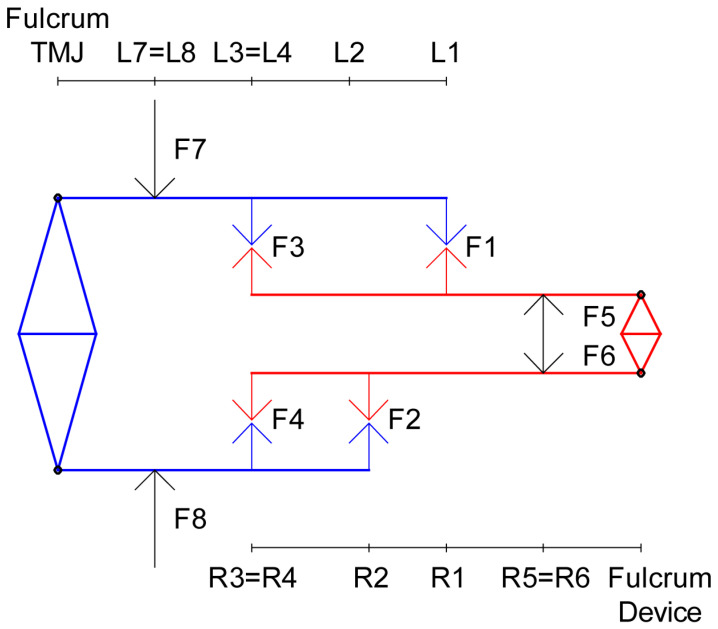
Schematic representation of masticatory system (blue) and device (red) model system. *F*7 and *F*8 are equal and opposite forces exerted by the masseter, *F*5 and *F*6 force recorded by the sensor. For simplicity, only two points of contact between the mouth and the device are considered for each arch: *F*1 and *F*3 for the upper part, *F*2 and *F*4 for the lower one.

**Figure A2:**
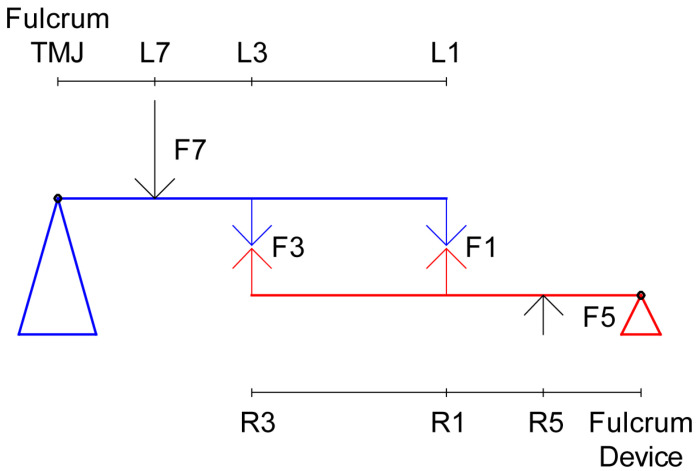
Schematic representation of upper component of the masticatory system (blue) and device (red) model system.

**Figure A3:**
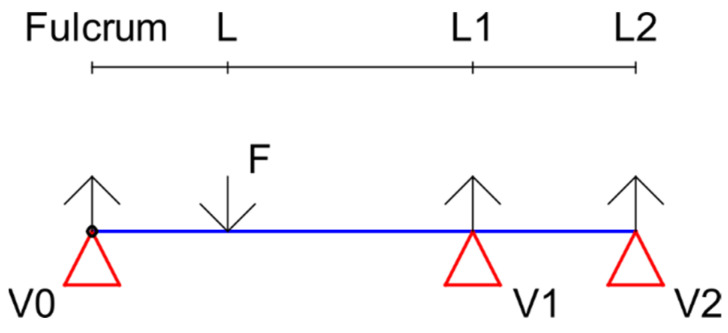
Hyperstatic beam representation.

**Figure A4:**
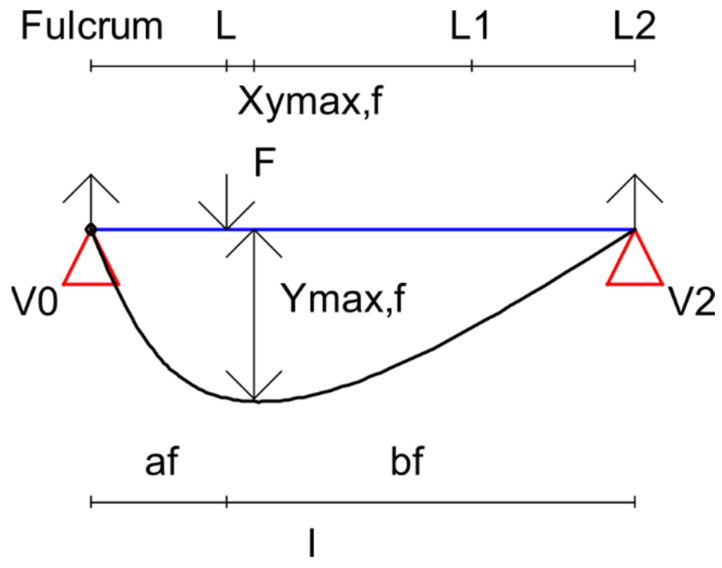
Representation of the hyperstatic beam without the constrain *L*1.

**Figure A5:**
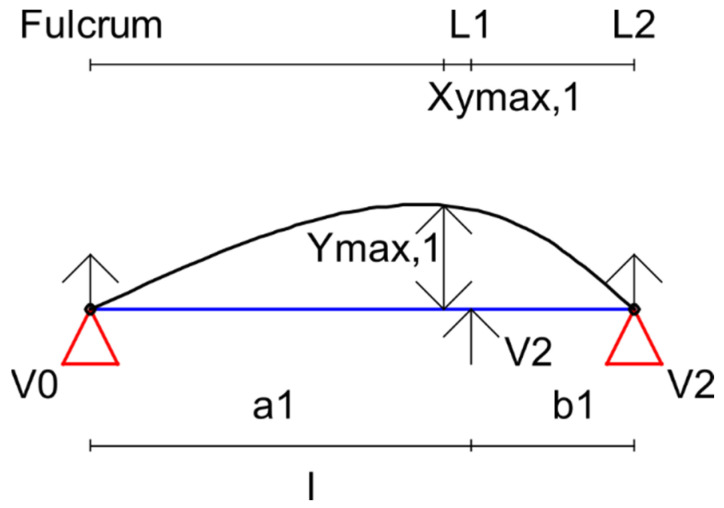
Representation of the hyperstatic beam without the force F.

**Figure A6:**
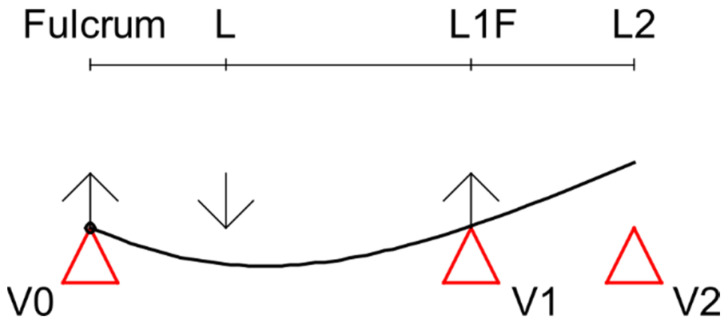
Representation of the effect superimposition analysis on the hyperstatic beam.

**Figure A7:**
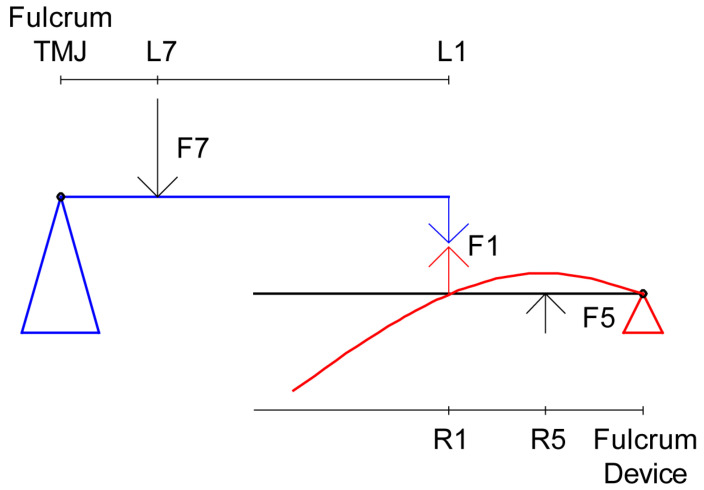
Representation of the single deformation acting only on the upper device fork plate.

**Figure A8:**
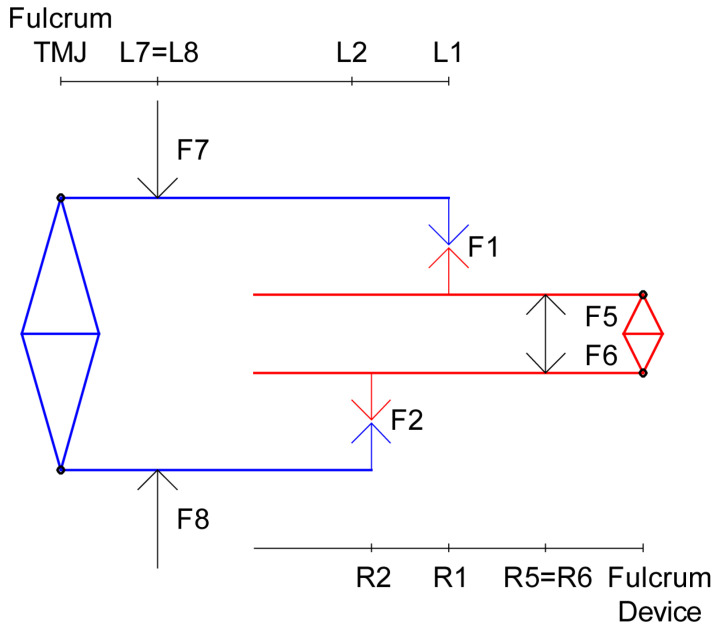
Representation of the masticatory system and the device with only one application point for each dental arc.

**Figure A9:**
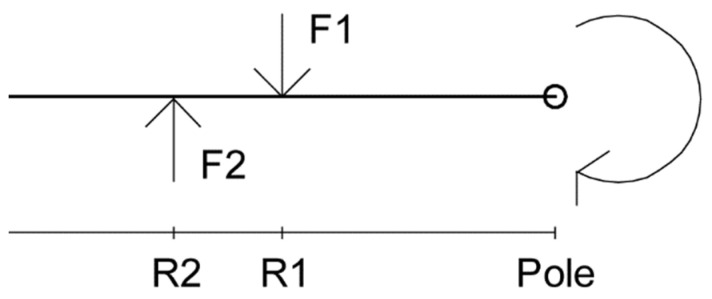
Representation of the masticatory system and the device neglecting internal forces.

**Figure A10:**
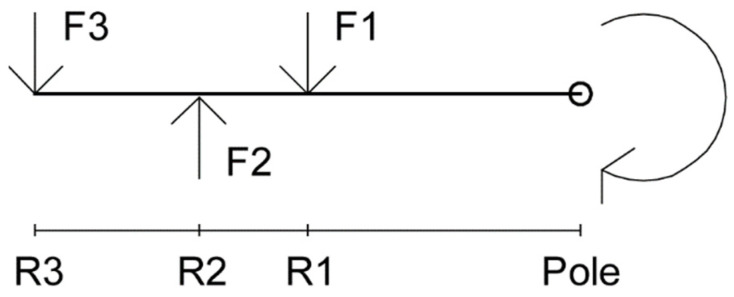
Representation of the masticatory system and the device neglecting internal forces and considering *R*3 as the moment unloading point.

## Data Availability

Calibration data are available on Zenodo, https://doi.org/10.5281/zenodo.6417196 (accessed on 30 May 2022).

## Supplementary Materials

The following supporting information can be downloaded at: https://www.mdpi.com/article/10.3390/ma15114000/s1, CAD drawing and stereolithography meshes S1: Sensor interface model and read-out components housing 3D CAD model and stereolithography meshes; Figure S1: Read-out components and circuitry scheme; Software S1: Arduino sketches and PC GUI C# Visual Studio project. Supplementary Materials are available on Zenodo, https://doi.org/10.5281/zenodo.6611805 (accessed on 30 May 2022).

## Appendix A

### Appendix A.1. Physical Model

In this section, the interaction between the masticatory system and the force measurement device can is described using a simple physical model. The equilibrium of momentum equations is used to characterize the device, which works as a second-order lever. Static methods have been applied to solve the problem, which shows an indeterminate solution. Through this model, the device can record the force applied at the most distant incisive teeth from the sensor, and thus the correct MBF estimation requires taking into account the participant’s dental arc morphology.

Figure A1 shows a simplified model of the masticatory system and the device. In this system, the masticatory system in blue and the force recording device in red are represented. The masticatory system can be described as a third-class lever with the effort (masticatory muscles force) between the fulcrum (condyle) and the load (teeth-device application points). Our device is modeled as a second-order lever with the load (force transducer) between the fulcrum and the effort (bite force).

In particular, equal and opposite *F*7 and *F*8 represent the force exerted by the masticatory muscles system, *F*5 and *F*6, instead of the force measured by the sensor. For simplicity, only two points of contact between the mouth and the device are considered for each arch: *F*1 and *F*3 for the upper part, *F*2 and *F*4 for the lower one. Table A1 reports the momentum equilibrium equations for both the device and the masticatory system. The method can be generalized to an arbitrary number of contact points.

**Table A1:** Momentum equilibrium equations for the device (left column) and the masticatory system (right column).

Momentum Equilibrium Equations	
Device	Masticatory System
Σ*M* = 0	Σ*M* = 0
(1) *M*5 = *M*1 + *M*3	(1) *M*7 = *M*1 + *M*3
(2) *M*6 = *M*2 + *M*4	(2) *M*8 = *M*2 + *M*4
(3) *M*5 = *M*6	(3) *M*7 = *M*8
*M*1 = *R*1·*F*1	*M*1 = *L*1·*F*1
*M*2 = *R*2·*F*2	*M*2 = *L*2·*F*2
*M*3 = *R*3·*F*3	*M*3 = *L*3·*F*3
*M*4 = *R*4·*F*4	*M*4 = *L*4·*F*4
*M*5 = *R*5·*F*5	*M*7 = *L*7·*F*7
*M*6 = *R*6·*F*6	*M*8 = *L*8·*F*8
*F*1·*R*1 + *F*3·*R*3 = *F*2·*R*2 + *F*4·*R*4	*F*1·*L*1 + *F*3·*L*3 = *F*2·*L*2 + *F*4·*L*4

If only the upper arches of the device and the masticatory system are considered (Figure A2), the model would be composed of two beams hinged at one end and resting on two supports (device-dental arc contact points). The force in both cases is applied between the hinge and the two supports, which will generate constraint reactions.

Each beam is considered separately, and since the number of unknown constraints is higher than the available equations, the problem is statically indeterminate (or hyperstatic) (Equation (A1)). However, while isostatic structures can be treated as rigid bodies, hyperstatic structures must be treated as deformable bodies to solve all the constraints. Therefore, both the masticatory system and the device as a hyperstatic beam whose load (representing the action exerted by the masticatory muscles) is applied between the fulcrum and the loads (device-teeth contact points) are considered (Figure A3). Hyperstatic structures require compatibility conditions and solid body deformation equations to be considered in addition to the static equilibrium equations for determining the internal forces and reactions. Therefore, the superposition effect principle [26] to the deformations of the redundant constraint, which is assumed to be the support in *L*1, was applied. The principle of superposition states that on a linear elastic structure, the combined effect of several loads acting simultaneously is equal to the algebraic sum of the effects of each load acting individually (Equation (A1)).

(A1)
$$\{\begin{array}{c} \Sigma VerticalForces=V0+V1+V2\\ \Sigma Ma=F\cdot L=V1\cdot L1+V2\cdot L2 \end{array}$$

### Appendix A.2. Principle of Superposition

1. Removing the constraint *L*1

If the constrain *L*1 is removed, a supported beam with a concentrated load is obtained (Figure A4). For the resolution, it is necessary to consider the differential equation of the elastic line [26].

If *af* > *bf*,

$$X_{Ymax,f}=\frac{\sqrt{l^{2}-bf^{2}}}{\sqrt{3}}\;Y_{max,f}=\frac{F\cdot bf\cdot \sqrt{{\left(l^{2}-bf^{2}\right)}^{3}}}{9\cdot \sqrt{3}\cdot E\cdot J\cdot l}$$

where *E* is the Young’s modulus and *J* is the second moment of area.

If *af* < *bf*, x-axes must be inverted:

$$\{\begin{array}{c} af^{\prime }=bf\\ bf^{\prime }=af \end{array}\;X_{Ymax,f\prime }=\frac{\sqrt{l^{2}-bf^{\prime 2}}}{\sqrt{3}}\;\mathrm{for}\;\mathrm{inverted}\;x-\mathrm{axes},\;X_{Ymax,f}=l-X_{Ymax,f\prime }\;\mathrm{for}\;\mathrm{original}\;x-\mathrm{axes},\;Y_{max,f\prime }=\frac{F\cdot bf\prime \cdot \sqrt{{\left(l^{2}-bf^{\prime 2}\right)}^{3}}}{9\cdot \sqrt{3}\cdot E\cdot J\cdot l}$$

2. Removing the force F

In this case, a supported beam with concentrated load is obtained (Figure A5).

If *a*1 > *b*1,

$$X_{Ymax,1}=\frac{\sqrt{l^{2}-b1^{2}}}{\sqrt{3}}\;Y_{max,1}=\frac{V1\cdot b1\cdot \sqrt{{\left(l^{2}-b1^{2}\right)}^{3}}}{9\cdot \sqrt{3}\cdot E\cdot J\cdot l}$$

If *a*1 < *b*1, x-axes must be inverted:

$$\{\begin{array}{c} a1^{\prime }=b1\\ b1^{\prime }=a1 \end{array}\;\;X_{Ymax,1\prime }=\frac{\sqrt{l^{2}-b1^{\prime 2}}}{\sqrt{3}}\;\mathrm{for}\;\mathrm{inverted}\;x-\mathrm{axes},\;X_{Ymax,1}=l-X_{Ymax,1\prime }\;\mathrm{for}\;\mathrm{original}\;x-\mathrm{axes},\;Y_{max,1\prime }=\frac{V1\cdot b1\prime \cdot \sqrt{{\left(l^{2}-b1^{\prime 2}\right)}^{3}}}{9\cdot \sqrt{3}\cdot E\cdot J\cdot l}$$

Since the displacements *Y_max,f_* and *Y_max_*_,1_ are in the opposite direction:

$$Y_{max,f}=Y_{max,1}$$

It is obtained:

 V1(F)

In our case:

$$V1=\frac{F\cdot bf\cdot \sqrt{{\left(l^{2}-bf^{2}\right)}^{3}}}{b1\cdot \sqrt{{\left(l^{2}-b1^{2}\right)}^{3}}}$$

Then, from the sum of the moments in the system of Equation (A1):

$$\Sigma Ma=F\cdot L=V1\cdot L1+V2\cdot L2\;V2=\frac{\left(F\cdot L-V1\cdot L1\right)}{L2}$$

Since:

F·L<V1·L1

Therefore:

V2<0

However, since *Y*2 is not strictly a support because it does not constrain the upward movement, the beam will flex (Figure A6). Therefore, the point of application of the force can be considered unique and, in particular, as the point closest to the force.

Despite the dental being modeled as a deformable beam, its lower deformability with respect to the device allows us to neglect it, only a deformation of the device is considered (Figure A7). Then, only *L*1 can be considered as a point of contact. The same approach can be replicated in the lower half of the system (Figure A8).

Since the distances of the upper and lower incisal teeth *R*1 and *R*2 are different, it is necessary to understand which of the two forces between *F*1 and *F*2 generates the moment recorded by the sensor (*M*5 or *M*6). Therefore, the internal forces are neglected, and then the device is modeled as a unique rigid beam (Figure A9).

Since the upper and lower forces are not applied along the same axis, the resultant unbalanced moment would generate a rotation. The direction of this moment will depend on the difference *R*1 − *R*2. However, since the real device does not actually rotate during the task, other constraints would compensate for the resultant moment. Therefore, the contact points *R*3 and *R*4 can be reconsidered as applied points for constraint forces, which balance that moment. Whether *R*1 < *R*2, the moment would have a clockwise direction and negative sign, which will unload on *R*3 (Figure A10).

ΣM=0M1+M3=M2F1·R1+F3·R3=F2·R2

In summary, the device in Figure A1 can be modeled as in Figure 4, where based on the difference between *L*1 and *L*2, an unload on *L*3 or *L*4 will occur.

If *R*1 < *R*2:

F4=0

Knowing the force recorded on the sensor *F*6, the force applied in *F*2 is obtained by summing the moments:

$$Ms=F6\cdot R6=F2\cdot R2\;F2=\frac{F6\cdot R6}{R2}$$

Then, from distance *L*2 (incisor-masticatory system fulcrum distance) and *L*8 (masticatory muscles-masticatory system fulcrum distance), the moment and, therefore, the force exerted by the masseter *F*8, can be calculated as a function of the force recorded at the sensor *F*6:

$$Mm=F8\cdot L8=F2\cdot L2=\frac{F6\cdot R6}{R2}\cdot L2$$

Otherwise, if *R*1 > *R*2:

$$F3=0\;Ms=F5\cdot R5=F1\cdot R1\;F1=\frac{F5\cdot R5}{R1}$$

Finally, from the distance *L*1 (incisor-masticatory system fulcrum distance) and *L*7 (masseter-masticatory system fulcrum distance), the moment and, therefore, the force exerted by the masseter *F*7, can be calculated as a function of the force recorded at sensor *F*5:

$$Mm=F7\cdot L7=F1\cdot L1=\frac{F5\cdot R5}{R1}\cdot L1$$
