# Supplementary material for: A Low-Cost Wireless Bite Force Measurement Device
Source: Materials (Basel). 2022 Jun 4;15(11):4000. doi: 10.3390/ma15114000 (PMC9182234; doi:10.3390/ma15114000)
Supplement: Supplementary file 1 [file materials-15-04000-s001.zip › materials-1717834-supplementary/Supplementary/S2 - Figure/Read-out components and circuitry scheme.pdf]

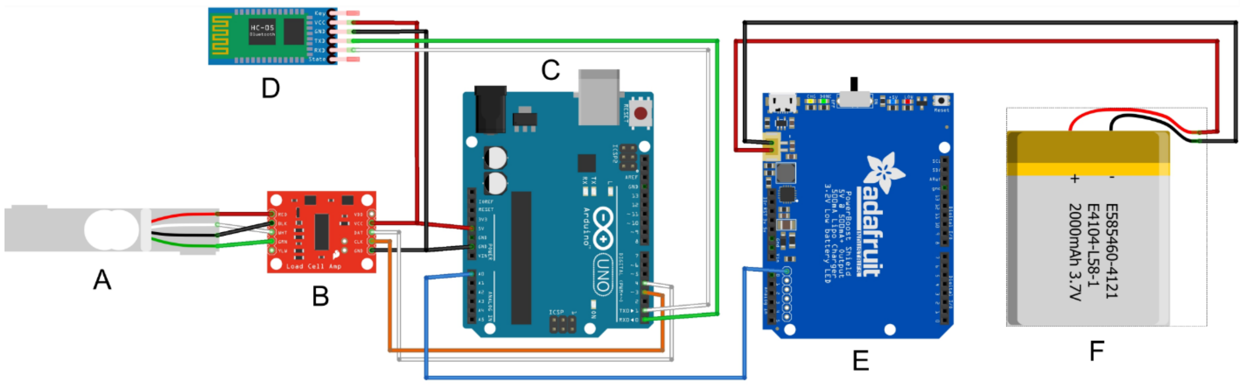

Figure S1. Read-out components and circuitry scheme. (A) and (B) show respectively a generic load cell and the connected signal amplifier HX711. (C) shows the Arduino UNO board connected to the Bluetooth module HC-05 (D), the battery charger shield (E) and the battery (F).
